# A Novel *CNTNAP2* Mutation Results in Abnormal Neuronal E/I Balance

**DOI:** 10.3389/fneur.2021.712773

**Published:** 2021-10-19

**Authors:** Ping Lu, Fengpeng Wang, Shuixiu Zhou, Xiaohua Huang, Hao Sun, Yun-Wu Zhang, Yi Yao, Honghua Zheng

**Affiliations:** ^1^Fujian Provincial Key Laboratory of Neurodegenerative Disease and Aging Research, Institute of Neuroscience, School of Medicine, Xiamen University, Xiamen, China; ^2^Jiangsu Province Hospital of Integrated Chinese and Western Medicine, Nanjing, China; ^3^Department of Functional Neurosurgery, Xiamen Humanity Hospital, Fujian Medical University, Xiamen, China; ^4^Department of Neurology, Xiamen University Hospital, Xiamen, China; ^5^Basic Medical Sciences, College of Medicine, Xiamen University, Xiamen, China; ^6^Shenzhen Research Institute, Xiamen University, Shenzhen, China

**Keywords:** spontaneous recurrent seizures, *CNTNAP2*, c.2329 C>G mutation, missense mutation, E/I balance

## Abstract

*CNTNAP2* (coding for protein Caspr2), a member of the neurexin family, plays an important role in the balance of excitatory and inhibitory post-synaptic currents (E/I balance). Here, we describe a novel pathogenic missense mutation in an infant with spontaneous recurrent seizures (SRSs) and intellectual disability. Genetic testing revealed a missense mutation, c.2329 C>G (p. R777G), in the *CNTNAP2* gene. To explore the effect of this novel mutation, primary cultured neurons were transfected with wild type homo *CNTNAP2* or R777G mutation and the morphology and function of neurons were evaluated. When compared with the vehicle control group or wild type group, the neurites and the membrane currents, including spontaneous excitatory post-synaptic currents (sEPSCs) and inhibitory post-synaptic currents (sIPSCs), in *CNTNAP2* R777G mutation group were all decreased or weakened. Moreover, the action potentials (APs) were also impaired in *CNTNAP2* R777G group. Therefore, *CNTNAP2* R777G may lead to the imbalance of excitatory and inhibitory post-synaptic currents in neural network contributing to SRSs.

## Introduction

In the central nervous system, the neuron network relies on effective signal transmission, especially the neurotransmitter transmission between neuron synapses. The balance between excitatory and inhibitory currents (E/I balance) became a hotspot of nervous system disorders' study in recent years (1–6). The relationship between inhibitory and excitatory synaptic transmission does not always remain stable, which may result in a lot of circuit dysfunctions and diseases, such as epilepsy, depression, anxiety, fragile X syndrome, Rett syndrome, Autism Spectrum Disorder (ASD), schizophrenia, and so on (7, 8). Additionally, studies suggest that gene alterations may be one of the underlying reasons (9). Gene deletion or mutation can change the excitability of neurons. For example, dysregulation of hippocampal inhibition was observed in *Cntnap2*^−/−^ mouse, which recapitulates the major features of ASD (10).

*CNTNAP2* (coding for protein Caspr2) gene locates in the long arm of the seventh autosomal chromosome (7q35) and encodes a protein named Caspr2 (Contactin-Associated-protein-like-2), which is a neuronal glycoprotein. Caspr2 is a transmembrane protein with a short intracellular fragment and a large extracellular component, which benefits interactions with other proteins, such as CNTN2, TAG-1, Kv1 channel, and protein 4.1B (11–13). *CNTNAP2* has been confirmed to be involved in several nervous system diseases including epilepsy, ASD, schizophrenia, language difficulties, and intellectual disability (14–21). Moreover, Caspr2 can cluster with the shaker Kv1.1 and Kv1.2 channels in the near lateral region of the flying junction of the axons with myelin sheath, involved in nerve conduction of myelin sheath axons (11, 22), and the process of neuron migration in mouse cortex (23). In *CNTNAP2* knockout mice, it was found that the activity of neural network was reduced, the dendrites were smaller, and the number of excitatory and inhibitory synapses was reduced, all of which may be caused by the effects of *CNTNAP2* deficiency on the neuronal synapses and dendrites (24).

Here, we describe a novel pathogenic *CNTNAP2* mutation in an 8-month-old infant who manifested spontaneous recurrent seizures (SRS) and intellectual disability. Whole exome sequencing revealed a novel pathogenic mutation, c.2329 C>G (p. R777G), in the *CNTNAP2* gene, causing the imbalance of excitatory and inhibitory post-synaptic currents in the neural network and contributing to SRSs.

## Materials and Methods

### Patient Reports and Ethics

An 8-month-old male infant had been presenting with infantile spasm since the age of 6 months. The seizure frequency was 2–3 clusters per day. He received standard anti-seizure medications (ASMs), including Valproate 30 mg/kg/d, Topiramate 1 mg/kg/d, and Lamotrigine 1 mg/kg/d. However, none of those treatments were effective. He had intellectual disability. He was unable to roll over or crawl alone. The history of his mother's pregnancy and delivery was normal. One of his collateral brothers suffered autism. We received approval from the Medical Ethics Committee of Fujian Medical University Xiamen Humanity Hospital using human materials (Permitted number HAXM-MEC-202000701-012-01). We also received informed consent for research from the participants or guardians.

### Genetic Procedures and Sanger Sequencing

Genetic testing was performed using targeted exome sequencing at Fuzhou Kingmed for Clinical Laboratory, China. DNA was extracted from blood of the patient using QIAamp Blood DNA Mini Kit (QIAGEN) and was purified by the magnetic bead method. DNA was subsequently amplified by PCR and connected with the upper joint sequence, captured, and purified by the TruSight one sequencing panel (Illumina Inc, USA). The obtained final DNA libraries were sequenced using a NextSeq500 sequencer (Illumina Inc, USA). Candidate mutations were verified by Sanger sequencing.

### Plasmid Construction

The human *CNTNAP2* cDNA was obtained from Han's Lab, School of Life Sciences, Xiamen University. Wild type pEGFP-N1-*CNTNAP2* plasmid was prepared by inserting coding sequence of the human *CNTNAP2* gene into the pEGFP-N1 vector. The *CNTNAP2* R777G mutation was obtained by PCR-based site-directed mutagenesis with c.2329 C>G.

### Transfection of Primary Neuron

According to the widely used protocol pioneered by Beaudoin (25), the primary neurons were isolated from the cortex of post-natal 0–1 day C57BL/6 mice in the Laboratory Animal Center of Xiamen University. All efforts were aimed to lessen animals' suffering. All animal experiments were performed in accordance with the protocols of the Institutional Animal Care and Use Committee at Xiamen University (Permitted number XMULAC20170255). After 13–14 days' mixture culture, the primary neurons were, respectively, transfected with plasmid of vehicle, wild type *CNTNAP2*, or *CNTNAP2* R777G mutation by Lipofectamine™ 2000 (Thermo) according to the manufacturer's instructions. Cells were then collected for further assay after 24 h.

### Quantification of Neurite Extension by Concentric Circle Intersection Count

For the transfected neuronal immunofluorescence observation, the primary transfected neurons with vehicle, wild type *CNTNAP2*, or *CNTNAP2* R777G mutation plasmid were fixed and captured with a Nikon T-P2 DIGITAL SIGHT microscope (Nikon, Japan). High-power field of at least five neurons per section from three independent experiments were selected for quantifying the number of intersections by Nikon NIS-Elements D 4.00.12 Viewer software. A series of concentric circles with radius increasing at 100 pixel (px) and spanning from 100 to 400 px range were plotted with the number of intersections against distance from the neuron center. The number of neurite intersections (branch) with each circle line along the distance from the neuron center was then manually quantified using a set of 12 neuron images in each group.

### Solution for Electrophysiology Recording

Artificial Cerebrospinal Fluid (ACSF): 120 mM sucrose, 64 mM NaCl, 2.5 mM KCl, 1.25 mM NaH_2_PO_4_, 26 mM NaHCO_3_, 10 mM d-glucose, 10 mM MgSO_4_, 0.5 mM CaCl_2_, pH 7.4, 290 mOsm.

Pipette Solution for recording spontaneous excitatory post-synaptic currents (sEPSCs) and spontaneous inhibitory post-synaptic currents (sIPSCs): 140 mM CsCH_3_SO_3_, 2 mM MgCl_2_ 6H_2_O, 5 mM TEA-Cl, 10 mM HEPES, 1 mM EGTA, 2.5 mM Mg-ATP, 0.3 mM Na-GTP, pH 7.4, 290 mOsm. The solution was filtered by 0.22 μm filter membrane after preparation in case the pipette was plugged.

Pipette Solution for recording action potentials (APs): 140 mM K^+^ gluconate, 4 mM NaCl, 0.1 mM CaCl_2_, 10 mM HEPES, 1.1 mM EGTA, 0.3 mM Na_2_-GTP, 2 mM Mg-ATP, pH 7.4, 290 mOsm. The solution was filtered by 0.22 μm filter membrane after preparation in case the pipette was blocked up during whole-cell patch recording.

Cortical neurons were recorded in whole-cell configuration 24 h after transfection. The extracellular solution was ACSF as described previously (26). Patch pipettes were pulled from borosilicate glass and fire-polished to a resistance of 3–5 MΩ. After pouring with pipette solution, neurons were voltage clamped at −70 mV for sEPSCs recording or at −0 mV for sIPSCs. Currents were recorded using pCLAMP 10.3 software with an Axopatch 700B amplifier (Molecular Devices: Axon CNS, MultiClamp 700B, Digidata 1440A, USA). To detect the events of post-synaptic currents, we set threshold of sEPSCs at 5 pA and sIPSCs at 10 pA, respectively. All the events were estimated as the 10%−90% rising time and decay time (ms). Recordings were filtered at 5 kHz and digitized at 20 kHz. The data were low-pass filtered using a 1 kHz cutoff and analyzed with Mini-Analysis 6.0.3 software (Synaptosoft, USA).

### Action Potential Recording

Quantitative electrical stimulations were applied to induce the neuronal APs. The input current increased by 10 pA in a stepwise manner. The amount of induced APs in different groups was recorded accordingly. The resting membrane potentials varied from −70 to −75 mV in this experiment. We set −70 mv as holding potential for recording sEPSC and −10 mv for sIPSC. The access resistance was also monitored during the experiment. The access resistance was usually 10–20 MΩ. If the access resistance was <10 MΩ or more than 20 MΩ, the data were excluded in our experiment.

### Statistical Analysis

All data were represented as mean ± standard error of mean (SEM). Statistical significance was determined by *one-way analysis of variance* (*ANOVA*) and by Bonferroni's *post-hoc* test by GraphPad Prism 6.0 statistical software. *p*<* 0.05* was considered significant.

## Results

### Identification of the Novel *CNTNAP2* R777G Mutation

**Figure 1:**
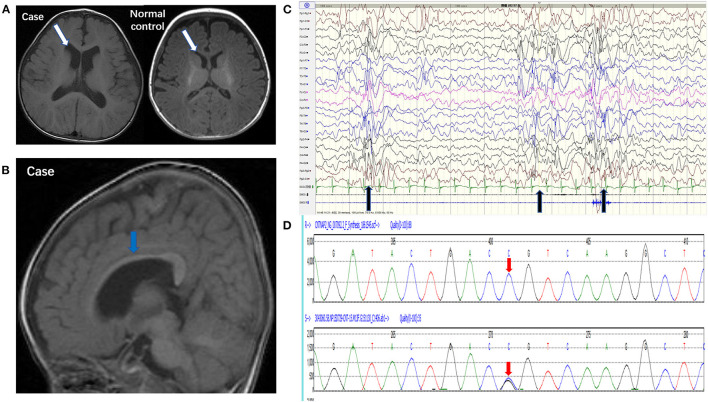
Pedigrees of the case carrying a *CNTNAP2* R777G mutation. **(A)** 3.0T MRI (axial T1) of the patient (left) and normal control (right) showed mild enlargement of ventricle (white arrow). **(B)** 3.0T MRI (sagittal T1) showed corpus callosum dysplasia (blue arrow). **(C)** EEG showed intermittent hypsarrhythmia and superimposed with epileptic discharges at bilateral parieto-occipital area (black arrow). **(D)** *CNTNAP2* DNA from peripheral blood leucocytes of the patient was analyzed by Sanger sequencing (the upper red arrow indicates normal base sequence and the lower red arrow indicates the c.2329 C>G mutation).

Brain MRI showed that the bilateral ventricular system was mildly enlarged (Figure 1A) and the corpus callosum was dysplastic (Figure 1B). Long-term EEG showed hypsarrhythmia background and frequently multifocal epileptic discharges (Figure 1C). Whole exome sequencing revealed a novel point mutation c.2329 C>G (p. R777G) in the *CNTNAP2* gene (Figure 1D).

### *CNTNAP2* R777G Decreases the Neurite Extension

**Figure 2:**
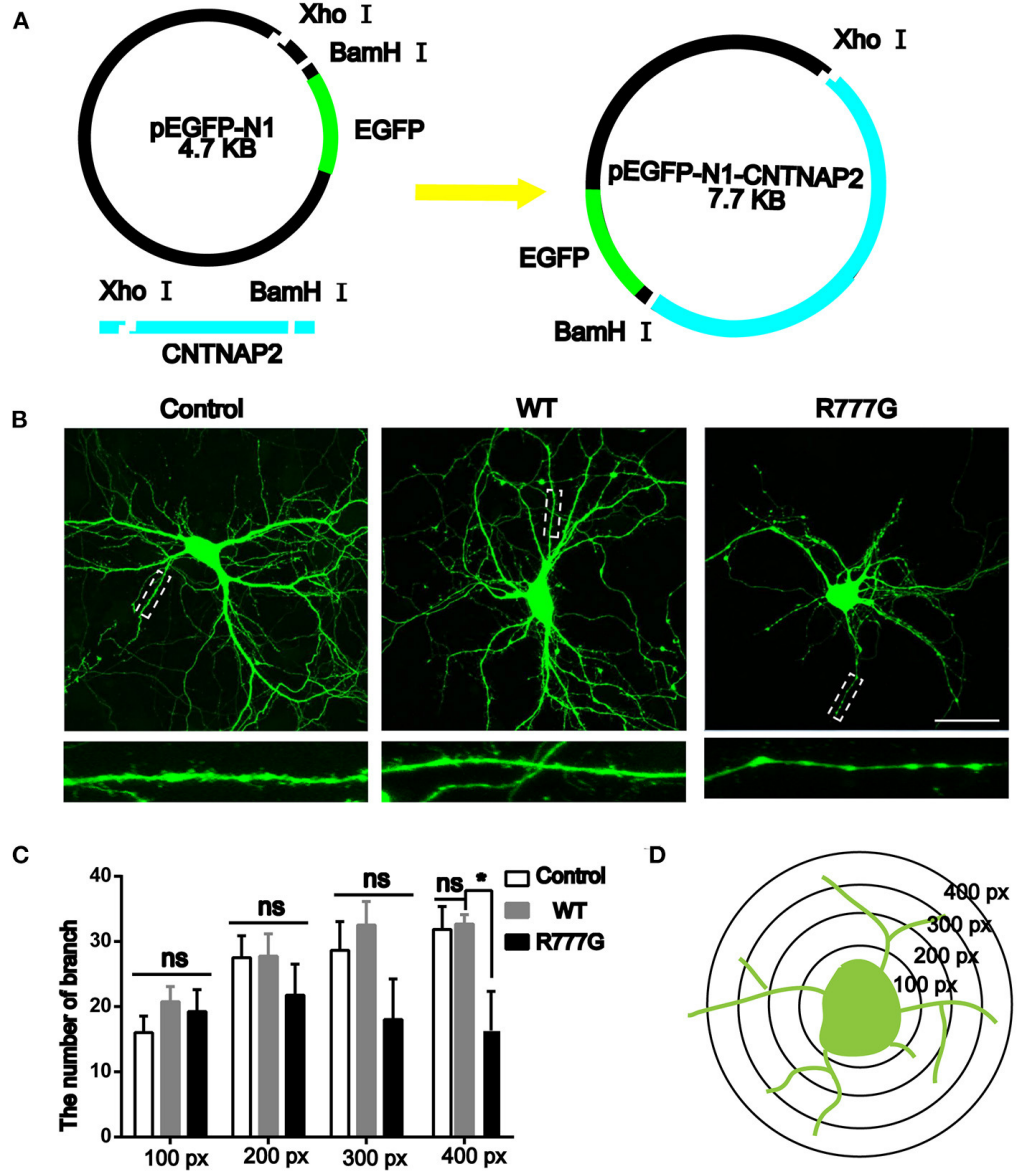
Effects of *CNTNAP2* R777G on the morphological change of primary cultured neurons. **(A)** Schematic diagram of the pEGFP-N1-CNTNAP2 plasmid, which can express GFP and Caspr2 simultaneously. **(B)** Representative fluorescent images of primary cultured neurons transfected with vehicle, wild type or *CNTNAP2* R777G plasmid, respectively. The enlarged images below are magnified views of the dotted square regions. **(C)** The number of neurites intersections in each circle was counted to obtain a statistical diagram of the number of dendrites of primary cultured neurons in different groups, with the radius of 100, 200, 300, and 400 px, respectively. **(D)** Schematic diagram of neurites intersections. Scale bar: 100 μm. *ANOVA* and Bonferroni's *post-hoc* test, *p < 0.05, ns, not significant.

Caspr2 is an adhesion molecule required for the formation of axoglial paranodal junctions surrounding the nodes of Ranvier (27). Therefore, it is probable that this novel mutation affects the function of Caspr2. To determine this, wild type pEGFP-N1-*CNTNAP2* or *CNTNAP2* R777G mutation plasmid was prepared (indicated in Figure 2A). Then, we overexpressed *CNTNAP2* R777G at a level comparable to that of wild type Caspr2 in primary cultured neurons. We found that the number of neurites in *CNTNAP2* R777G group was decreased when compared with wild type (WT) group (Figure 2B). The dendrite branches were also reduced with an increasing distance from neuron soma, and the mutation neuron dendrites were sparse, especially distal dendrites (Figure 2C). Figure 2D indicated the schematic diagram of neurite intersections. These results indicate that *CNTNAP2* R777G decreases the neurite extension.

### *CNTNAP2* R777G Affects Post-synaptic Currents of Neurons

**Figure 3:**
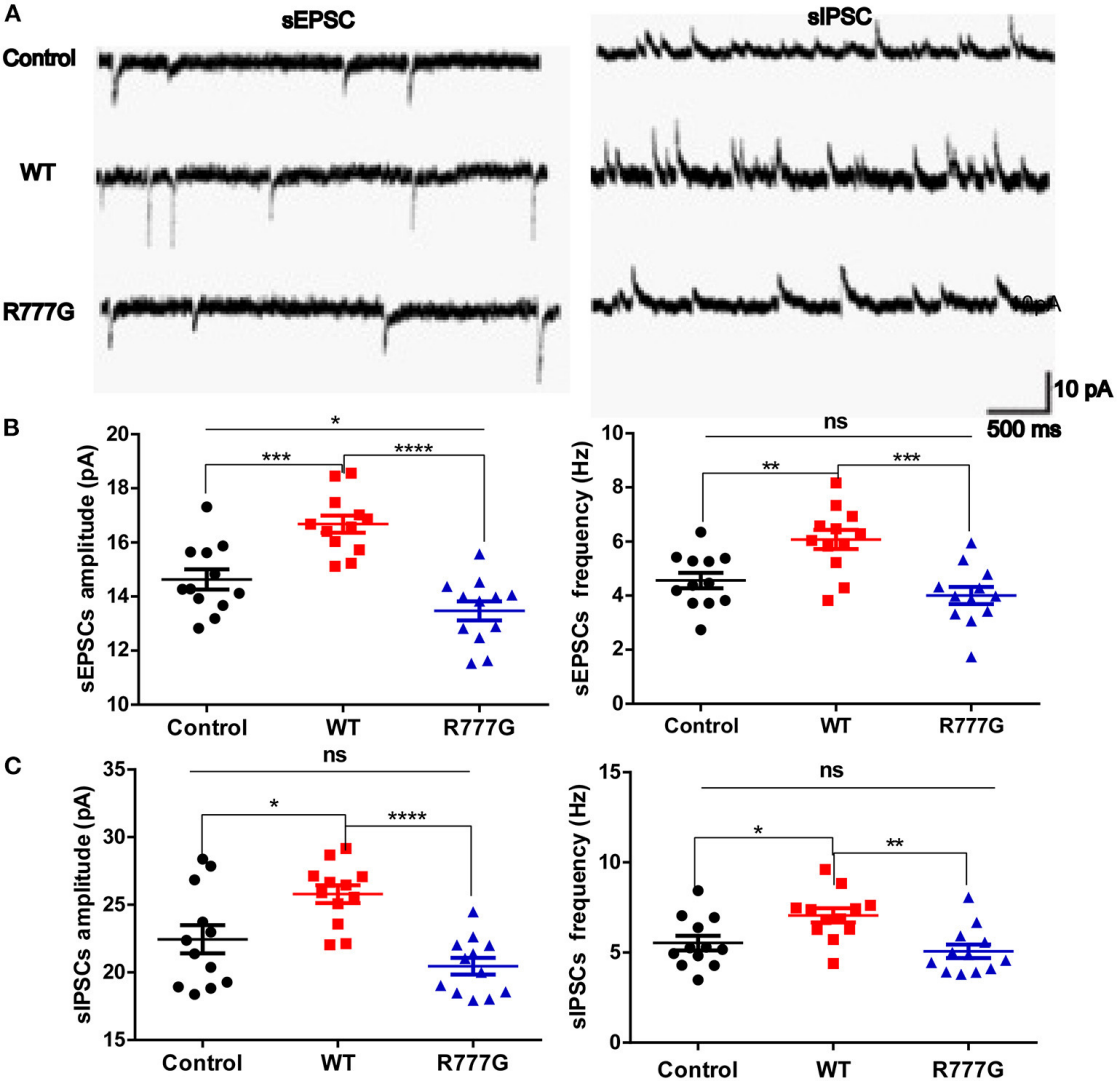
*CNTNAP2* R777G affects the post-synaptic currents of neurons. **(A)** The trace of sEPSCs or sIPSCs. **(B)** The amplitude and frequency of sEPSC in different groups. **(C)** The amplitude and frequency of sIPSC in different groups. *n* = 8 for each group. *ANOVA* and Bonferroni's *post-hoc* test, *p < 0.05, **p < 0.01, ***p < 0.001, ****p < 0.0001.

Given that loss of Caspr2 contributed to the aggregates of cytoplasmic glutamate receptor (28, 29), we wondered whether the neuronal excitability was disrupted by *CNTNAP2* R777G mutation. We then employed primary cultured neurons with the whole cell patch to analyse the excitability of cells. We variably clamped the membrane potential to differentially record sEPSCs and sIPSCs, which are rough methods of measuring neuron excitability (30, 31). We found that both the amplitude and frequency of sEPSCs in *CNTNAP2* WT group were increased whereas *CNTNAP2* R777G group showed lower amplitude of sEPSCs when compared with control group (Figures 3A,B), suggesting the important role of Caspr2 in neural excitatory activity. One study reported that loss of Caspr2 would increase post-synaptic excitatory responses (32). In contrast, the novel R777G mutation might negate the function of Caspr2 and compromise its physiological function. However, the amplitude and frequency of sIPSC in Caspr2 WT group also increased compared with control group (Figure 3C). These results might be explained as a compensatory change in *CNTNAP2* WT group in response to the increased excitability of neurons. Therefore, these results indicate that *CNTNAP2* R777G lost the function of maintaining the normal E/I balance.

### *CNTNAP2* R777G Mutation Impairs the Action Potential of Neurons

**Figure 4:**
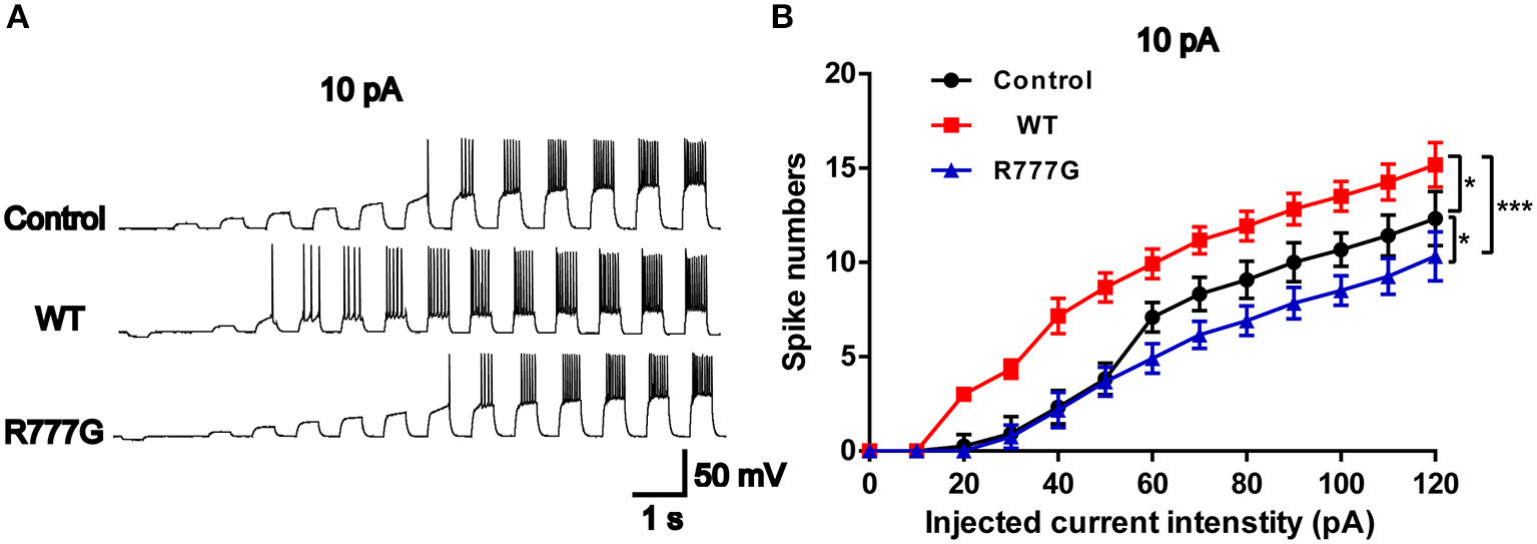
*CNTNAP2* R777G impairs the action potentials of neurons. **(A)** The sequential sweeps of APs in different groups evoked with different intensity of injected currents. **(B)** Evoked action potentials were quantified to compare the neuronal excitability in different groups. *n* = 4 or 5 for each group. *ANOVA* and Bonferroni's *post-hoc* test, *p < 0.05, ***p < 0.001.

Action potentials is always considered as one of the indicators of neural excitability. All the receptor-mediated sEPSCs and sIPSCs belong to partial synaptic events so that we can evaluate the neural excitability through APs induced by the integration of many synaptic activities (33). Action potentials resulted from transient changes in the permeability of the axon membrane to sodium and potassium ions (34). We recorded neural APs induced by exogenous current and got a cluster of APs when the neurons were stimulated by inputted currents (Figures 4A,B). In the current experimental conditions, the tested neurons exhibited a tonic pattern whereas the phasic pattern of APs was not noted in the tested neurons. As expected, neurons in *CNTNAP2* WT group induced more APs than those in other groups, further proving that Caspr2 participate in the formation of neuronal excitation (Figures 4A,B). In addition, APs threshold was detected in three groups, and neurons in *CNTNAP2* R777G group showed the highest APs threshold with the fewest number of APs compared with those in other groups (Figures 4A,B). These results suggest that *CNTNAP2* R777G mutation impairs the AP of neurons.

## Discussion

In this study, we identified a novel pathogenic R777G mutation in *CNTNAP2* gene in an atypical infant with SRSs. This *CNTNAP2* R777G mutation caused the imbalance of sEPSCs and sIPSCs in the neural network, contributing to SRSs, which was consistent with the literature on *CNTNAP2* knockdown experiments. Furthermore, as a susceptible gene of epilepsy, *CNTNAP2* mutation or deletion results in the break of E/I balance in neuronal network.

Caspr2 is a member of the axon superfamily that promotes intercellular interactions in the nervous system. Our finding that *CNTNAP2* R777G decreases the neurite extension is consistent with other researchers' findings that deletion of Caspr2 resulted in the deficit in dendrite arborization and reduction in the dendritic length and branching of interneuron (35).

Axon and dendrite terminals play key roles in synaptic function in the neural network and the receptor-mediated membrane currents are indicators of neuronal excitability. GABAergic synapses reside on dendritic shafts, soma, and axon initial segments in the formation of predecessor axon-dendrite contacts (36). On the contrary, glutamatergic synapses form almost exclusively on dendritic spines (37). Some researchers also observed that depletion of Caspr2 in neurons decreased synaptic strength in a cell-autonomous fashion, impaired terminal dendrites and spine development, and suppressed neural network activity (24). Additionally, Caspr2 plays an important role in the development and activity of normal neuronal network whereas mutation in *CNTNAP2* gene can disorganize normal Caspr2 functions.

Researchers recently observed that deletion of Caspr2 resulted in the reduction of the amplitude of α-amino-3-hydroxy-5-methyl-4-isoxazole propionate receptor (AMPAR) and N-methyl-D-aspartate receptor (NMDAR)-mediated EPSCs and the amplitude of γ-aminobutyric acid type A (GABA_A_) receptor-mediated IPSCs (24, 38, 39). In other words, the synaptic transmission was reduced in *CNTNAP2-*deficient neurons. The synaptic transmission of GABAergic interneuron was also decreased in *CNTNAP2* knockout mice (23). It is still unclear what the reason for the sEPSCs alterations in *CNTNAP2* R777G group is, which may result from abnormal synapses or decreased neurites induced by *CNTNAP2* R777G in neurons. Therefore, these results indicate that *CNTNAP2* R777G lost the function of maintaining the normal E/I balance. Although Caspr2 was recently reported to be expressed in both excitatory and inhibitory synapses and effects of Caspr2 depletion were found on excitatory and inhibitory currents (38, 39), the specific types of neurons *CNTNAP2* R777G affects needs to be further explored.

Given that Caspr2 can cluster with Kv1.1 and Kv1.2 channels involved in the nerve conduction of myelin sheath axons (11, 22), there is a possibility that this result is due to a change in voltage-gated ion channels, such as Kv1.1 or Kv1.2. *CNTNAP2* R777G neurons showed the highest APs threshold with the fewest number of APs compared with other groups, which may result in circuit dysfunctions and diseases. However, how this *CNTNAP2* R777G mutation contributes to SRSs *in vivo* remains elusive.

In conclusion, this study provides clinical and experimental data to demonstrate a novel pathogenic R777G mutation in *CNTNAP2* gene in an atypical infant with SRSs. Nevertheless, the findings of the present study are limited. Without a large family showing seizure and enough gene samples from family members, it is difficult to demonstrate that *CNTNAP2* R777G was fully responsible for this disease. Additionally, the overexpression system in this study is artificial. It is interesting to investigate whether *Cntnap2* R777G mutant mouse develops SRSs in future work.

## Data Availability Statement

The datasets presented in this article are not readily available due to privacy restrictions. Requests to access the datasets should be directed to the corresponding author.

## Ethics Statement

The studies involving human participants were reviewed and approved by Medical Ethics Committee of Xiamen University for our experiments using human materials. We also received informed consent for research from the participants or guardians. Written informed consent to participate in this study was provided by the participants' legal guardian/next of kin. All animal experiments were performed in accordance with the protocols of the Institutional Animal Care and Use Committee at Xiamen University.

## Author Contributions

HZ conceived and designed the study. PL, FW, and SZ performed the experiments and analyzed the data. PL, FW, and HZ wrote the paper. YY provided the information on the case. XH, HS, and Y-WZ coordinated the study and provided technical assistance. All authors reviewed the results and approved the final version of the manuscript.

## Funding

This work was supported by grants from the Natural Science Foundation of Fujian Province of China (2018D0022 and 2020J01014). This study was also supported by grants from the National Natural Science Foundation of China (81771164, 91949129, 81771377, 81471160, and U1705285).

## Conflict of Interest

The authors declare that the research was conducted in the absence of any commercial or financial relationships that could be construed as a potential conflict of interest.

## Publisher's Note

All claims expressed in this article are solely those of the authors and do not necessarily represent those of their affiliated organizations, or those of the publisher, the editors and the reviewers. Any product that may be evaluated in this article, or claim that may be made by its manufacturer, is not guaranteed or endorsed by the publisher.
